# Dissipation and Dietary Risk Assessment of Prochloraz in Strawberries under Greenhouse Conditions

**DOI:** 10.3390/molecules28227498

**Published:** 2023-11-09

**Authors:** Hui Wang, Jian Sun, Qingju Liu, Cheng Li, Yunxia Luan

**Affiliations:** 1 Institute of Quality Standard and Testing Technology, Beijing Academy of Agriculture and Forestry Sciences, Beijing 100097, China; wangh@iqstt.cn (H.W.);; 2 Institute of Forestry and Pomology, Beijing Academy of Agriculture and Forestry Sciences, Beijing 100097, China; sjroad@126.com

**Keywords:** dissipation, metabolites, prochloraz, risk assessment, strawberry

## Abstract

Prochloraz and its metabolites in strawberries have not been determined until now. Meanwhile, few reports in the literature have concerned the dissipation behavior and risk assessment of prochloraz and its metabolites in strawberries under greenhouse conditions in Beijing. A method for the determination of prochloraz and its metabolites in strawberries was developed using QuEChERS in combination with ultra-performance liquid chromatography–tandem mass spectrometry (UPLC-MS/MS). Prochloraz and its metabolites recovered from strawberries were present in concentrations of 73.06% to 116.01%, their RSDs ranged from 1.12% to 9.17%, and their limits of detection ranged from 0.1 to 1 μg kg^−1^. Then, a study was conducted on the dissipation of prochloraz in strawberries under greenhouse conditions. The dissipation of prochloraz in strawberries followed the first-order kinetic equation, and its half-life was 8.06 days. The health risk associated with prochloraz in strawberries was evaluated using the target hazard quotient (THQ) method and EFSA PRIMo model. The results showed that the THQ values, %ARfD values, and %ADI values were less than 1. These results indicate that no health concerns of prochloraz are associated with the consumption of the studied strawberries. The government can use the results of this study to support the establishment of a maximum residue level for prochloraz in strawberries.

## 1. Introduction

Prochloraz is a highly effective and broad-spectrum imidazole fungicide used in crop growth to provide preventive protection and curative functions. In addition, it is used for the storage and preservation of fruits and vegetables [1,2,3]. Prochloraz inhibits the activity of lanosterol 14 α-demethylase (CYP51A1), which is necessary for the generation of fungal ergosterol [4]. Ergosterol is an essential component in the fungal cell membrane. Therefore, prochloraz is widely applied to prevent and cure diseases of oil crops, cereals, fruits, vegetables, and cash crops caused by sclerotinia, fusarium, powdery mildew, anthrax, and other pathogenic bacteria [5,6,7,8]. Despite its germicidal properties, prochloraz can cause dysplasia due to its disruptive endocrine properties [9]. Furthermore, the toxicity of trichlorophenol, which is one of the metabolites of prochloraz, has significantly increased [10,11]. Therefore, studies on the determination and dissipation of prochloraz and its metabolites are of great significance for protecting consumers’ health.

**Figure 1:**
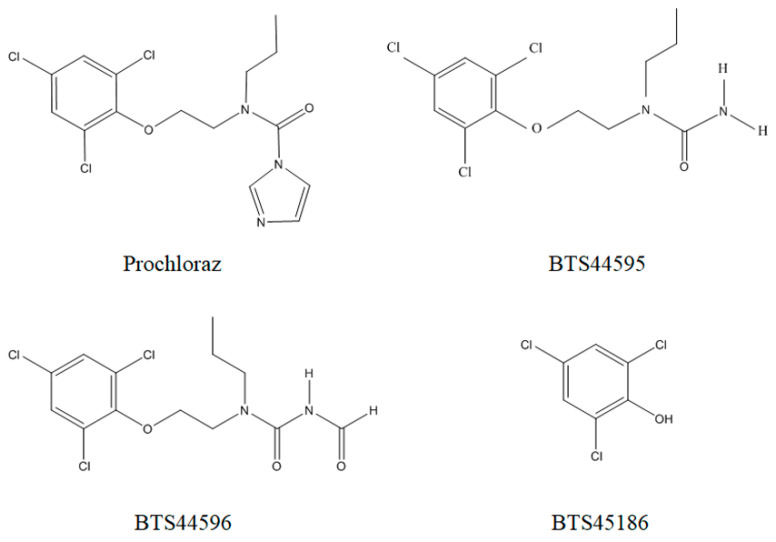
Structural formulas of prochloraz and its metabolites.

In plants, prochloraz breaks its imidazole ring and produces its main metabolites (Figure 1), including *N*′-formyl-*N*-propyl-*N*-[2-(2,4,6-trichlorophenoxy)ethyl]urea (BTS44596) and *N*-propyl-*N*-[2-(2,4,6-trichlorophenoxy)ethyl]urea (BTS44595). These two compounds can break the carbon–oxygen bond and degrade into 2,4,6-trichlorophenol (BTS45186), which is a free and conjugated metabolite. There are also traces of 2–(2,4,6-trichlorophenoxy)–acetic acid [12,13,14,15]. It was reported that BTS45186 might adversely affect humans’ nervous and respiratory systems, resulting in chronic bronchitis, cough, and altered lung function [2,16]. The structural stability and durability of BTS45186 are of great concern because of its high toxicity and carcinogenicity. At present, organic solvent extraction and QuEChERS are the main methods applied to extract prochloraz and its metabolites in bananas, cucumbers, carrots, and other agricultural products [13,17]. An extraction solution was purified using Bondesil C18, primary secondary amine (PSA), graphitized carbon black (GCB), and other adsorbents, and then, the samples were determined using GC-MS, LC-MS/MS and other analytical instruments. GC-MS was applied to detect prochloraz in orange juice with detection and quantification limits of 3.2 and 10.8 μg kg^−1^, respectively [18]. Fan et al. used LC-MS/MS to determine prochloraz in *Fragaria* and *Myrica rubra*, and the LOD was ≤3 μg kg^−1^ [19]. Tian et al. used HPLC-MS/MS to determine prochloraz and its metabolites in grapes, red wine, and pomace, and the LOQ values ranged from 1 to 50 μg kg^−1^ [20]. Radio-UPLC-HRMS was applied to analyze prochloraz and 2,4,6-trichlorophenol in Rapeseed Oil with an LOQ of 5 μg kg^−1^ [21]. Nevertheless, no methods have been reported for determining prochloraz and its metabolites in strawberries. Therefore, the present study established a method to measure prochloraz and its metabolites in strawberries.

Until now, prochloraz dissipation has been reported in previous research. It is important to note that the dissipation rate of prochloraz is affected by various factors. Its half-life varies with its matrix. A study reported that the dissipation half-life of prochloraz was 8.89–18.02 days and 5.79–12.38 days in soil and apple, respectively [12]. The half-life of prochloraz is also affected by sprayed concentration. At 25 °C, the half-life of prochloraz was 13.9 days when the concentration of prochloraz was 0.450 g a.i./L, and its half-life was 8.8 days when the concentration of prochloraz was 0.225 g a.i./L [22]. It was found that the half-life of pesticides was affected by location [23], which might be due to the diverse environmental factors in different places. However, limited reports on the dissipation behavior and risk assessment of prochloraz in strawberries under greenhouse conditions have been published.

Therefore, this study aims to establish an efficient, sensitive, accurate, and reliable analytical method based on QuEChERS combined with UPLC-MS/MS to determine prochloraz and its metabolites simultaneously. In this study, the dissipation kinetics of prochloraz in strawberries was studied under greenhouse conditions in Beijing, and the dietary risk was assessed. Based on the results of this research, supporting data could be provided for setting a maximum residue limit (MRL) of prochloraz in strawberries in China to ensure food safety.

## 2. Results and Discussion

### 2.1. Optimization of Chromatographic and Mass Spectrometric Conditions

Special consideration was given to the optimization of the MS/MS parameters to enable highly sensitive and reliable quantification for prochloraz and its metabolites. Four target compounds were prepared in a 100 μg L^−1^ single standard solution. After the liquid phase flow rate was set to 0.3 mL min^−1^, the standard was injected into the mass spectrum directly. Then, the tuning function of Masslynx 4.1 software was applied to find parent and daughter ions of the target compounds. The optimal mass spectrum parameters of each ion were fixed by optimizing the cone voltage, collision energy, retention time, etc., as shown in Table S1. BTS45186 was detected in ESI- ionization mode, and the other chemicals were detected in ESI+ ionization mode.

Optimization of the chromatographic column and mobile phase is an effective means of separating and characterizing prochloraz and its metabolites [24,25,26]. An ACQUITY UPLC BEH C18 column (2.1 mm × 100 mm, 1.7 μm) and ACQUITY UPLC HSS T3 column (2.1 mm × 100 mm, 1.8 μm) were selected for separating prochloraz and its metabolites in this study. It was found that the ACQUITY UPLC HSS T3 chromatographic column showed a better separation effect on the target chemicals compared with ACQUITY UPLC BEH C18 by comparing retention time, ionization efficiency, and other factors (Figure S1). Hence, the ACQUITY UPLC HSS T3 column was chosen for subsequent experiments. Compared with methanol, the separation effect of compounds was better when the organic mobile phase was acetonitrile (Figure S2). The chemical separation effect and detection sensitivity were best when ultrapure water with 5 mM ammonium acetate and acetonitrile was used. Thus, this mobile phase was applied for further study.

### 2.2. Method Validation

The linearity, sensitivity, precision, and accuracy of the method were validated according to European Commission SANTE/11312/2021 [27]. A matrix-matched calibration curve was used to adjust for possible matrix interference on analyte quantification. The proposed method exhibits excellent linearity at a concentration of 1–100 μg kg^−1^. The study results indicated that the method was linear, and the determination coefficient R^2^ was higher than 0.9969. LOD was defined as the concentration of analyte detectable at a signal-to-noise ratio of 3:1. The LOD of prochloraz, BTS44595, BTS44596, and BTS45186 in strawberries was 0.1 μg kg^−1^, 0.1 μg kg^−1^, 0.1 μg kg^−1^, and 1 μg kg^−1^, respectively (Table 1). The lowest spiked concentration of the target analyte in the matrix was taken as the LOQ. There were no unified limits on prochloraz and its metabolites. The MRL of prochloraz and its metabolites in strawberries according to EU standards is 0.03 mg kg^−1^ [1]. There is no MRL regulation in China’s national standards. The LOD (or LOQ) of this method is far lower than the maximum residue limit in strawberries. Addition and recovery tests at low, medium, and high concentration levels were conducted within the range of 10–100 μg kg^−1^. The results showed that the recovery of prochloraz, BTS44595, BTS44596, and BTS45186 in strawberry samples ranged from 73.06% to 116.01%. Precision was expressed as the relative standard deviation (RSD) of five replicate analyses on the same day, while within-laboratory reproducibility was determined on five consecutive days by three operators at three fortification levels. The RSDs were 1.12–9.17%, which indicates that this method could be used to determine prochloraz and its metabolites in strawberries. The MRM images of prochloraz and its metabolites are shown in Figure S1.

In summary, the validation of this method (Table 2) demonstrated satisfactory results. A few established methods for analyzing prochloraz, and one or two of its metabolites, have been reported so far [18,28]. Furthermore, a method of determining prochloraz and its three metabolites was developed in this study. Compared with previous methods [13], the separation of the compounds in UPLC was better, especially in terms of the interference between two compounds with similar structures flowing out at the same retention time being avoided, and the results are accurate and reliable.

### 2.3. Dissipation Behavior of Prochloraz in Strawberries

**Figure 2:**
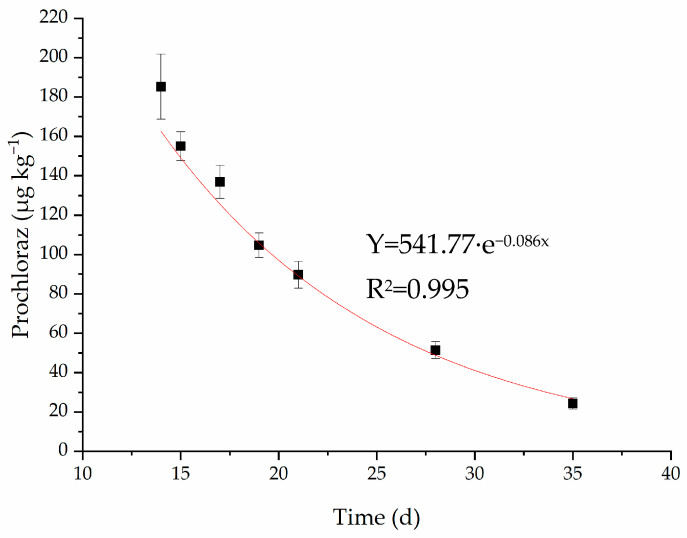
Dynamic curve of dissipation of prochloraz in strawberries.

Spraying in the field experiment for prochloraz EC was carried out three times over a 35 d period. The first and second sprayings were insufficient to fit the curve. Therefore, the dissipation of prochloraz in strawberries under greenhouse conditions after the third spraying was analyzed. The kinetic equation of prochloraz dissipation in strawberries is shown in Figure 2. The residual concentrations of prochloraz decreased with time, and its dissipation behavior was in line with the first-order kinetic equation. The regression coefficient R^2^ of the dissipation equation was 0.995. The half-life of prochloraz in strawberries was 8.06 days according to Formula (2). This result is similar to the half-life of prochloraz obtained in pear peel [22].

**Figure 3:**
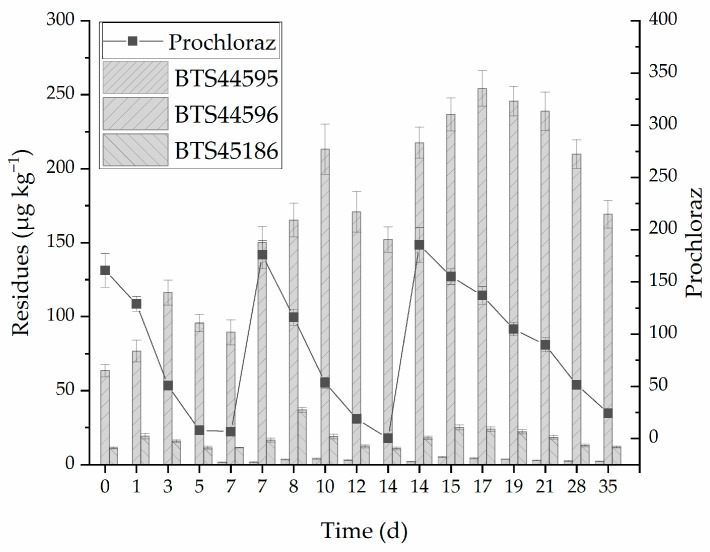
Dissipation of prochloraz and its metabolites in strawberries.

In this study, the spraying of 25% prochloraz EC at different concentrations (0.015 kg a.i./ha and 0.01 kg a.i./ha) in strawberries was conducted under greenhouse conditions. After spraying, prochloraz in strawberries dissipated, while BTS44595, BTS44596, and BTS45186 were generated, as shown in Figure 3. A downward trend was observed in prochloraz after spraying. After the third spraying, the prochloraz residue (*C*_0_) was 185.3 μg kg^−1^. On the 35th day, the concentration reduced to 24.3 μg kg^−1^. The main metabolite of prochloraz is BTS44596. After prochloraz EC spraying, the BTS44596 concentration showed an increasing trend in the first three days; then, it decreased. Its residue was 169.2 μg kg^−1^ on the 35th day. The concentration of BTS44595 residue was lower than the LOD within the first 5 days after spraying, and the concentration was 1.7 μg kg^−1^ on the first day after the second spraying. The compound concentration reached the highest value of 5.1 μg kg^−1^ on the 15th day, and the residual concentration was 2.2 μg kg^−1^ on the 35th day. The concentration of BTS45186 decreased to 11.9 μg kg^−1^ on the 35th day. The sum of prochloraz, BTS44595, BTS44596, and BTS45186 residues was 195.7 and 101.6 μg kg^−1^ in the high- and low-concentration groups, respectively. The residue concentrations were higher than the 30 μg kg^−1^ specified in the EU standards for residue.

The dissipation of prochloraz metabolites in strawberries is a dynamic process that is affected by their formation rate and dissipation rate. In strawberries, residual concentrations of prochloraz metabolites decrease when the generation rate is lower than the decomposition rate, and vice versa. The formation and degradation rates of chemicals in crops are affected by many factors, such as ambient temperature, humidity, and light [3,29,30]. Therefore, further study of the dissipation mechanism of prochloraz and its metabolites is necessary.

### 2.4. Dietary Risk Assessment of Prochloraz in Strawberries

The prochloraz concentration of the low-concentration spraying group on the 35th day was used for analyzing the dietary risk assessment. The dietary risk of prochloraz and its metabolites in strawberries is related to the consumer’s age, daily intake, and body weight, and pesticide residues, as shown in Table 3. The EDI values of prochloraz and its metabolites in Chinese children and adults were 2.0 × 10^−4^ and 1.5 × 10^−4^, respectively. In the second approach (EFSA PRIMo), the ARfD and ADI of prochloraz were set at 0.025 and 0.01 mg/kg bw/day, respectively, by EFSA [31]. The calculated %ARfD values of prochloraz for children and adults are 4.22% and 3.71%, respectively. And the calculated %ADI values of prochloraz for children and adults are 2.50% and 1.16%, respectively. All the values were less than 1, indicating no obvious risk of dietary intake of prochloraz and its metabolites in strawberries.

## 3. Materials and Methods

### 3.1. Reagents and Standards

Prochloraz and its metabolites BTS44595, BTS44596, and BTS45186 were purchased from Dr. Ehrenstorfer (Augsburg, Germany); LC-MS-grade methanol, acetonitrile, ammonium formate, and ammonium acetate were purchased from Thermo Fisher Scientific (Waltham, MA, USA); and analytical-grade magnesium sulfate and sodium chloride were purchased from Aladdin (Shanghai, China).

### 3.2. Greenhouse Experiment

#### 3.2.1. Dynamic Dissipation and Final Residue Test of Prochloraz in Strawberries

The experiment was carried out at the Tongzhou Yujiawu strawberry experimental base of the Institute of Forestry, Beijing Academy of Agriculture and Forestry Sciences, in 2021–2022. The trials were conducted according to the “Guidelines for the testing of pesticide residues in crops NY/T 788-2018” [32]. We sprayed 25% prochloraz emulsifiable concentrate (EC) (Rosi Chemical Co., Ltd., Yueqing, China) on strawberries in the greenhouse. The experiment was divided into a high-concentration group (1.5 times the recommended maximum application concentration, 0.015 kg a.i./ha), a low-concentration group (recommended maximum application concentration, 0.01 kg a.i./ha), and a control group (sprayed with water). The high-concentration group was used to study the dissipation of prochloraz in strawberries, while the low-concentration group was used to study the residue concentration of prochloraz in strawberries. The spraying of prochloraz EC was repeated on three plots. All the treated sample plots and reference plots were arranged randomly. Prochloraz was sprayed on the strawberries when the fruits were half the size of mature fruits three consecutive times at 7 d intervals. Sampling was conducted after 2 hours (0 day), 1 day, 3 days, and 5 days, respectively after each spraying, and the sampling time was extended by 14 days after the third spay compared to the previous two sampling time. Meanwhile, sampling was conducted after 7, 14, and 21 days after the third spraying. Strawberry samples were collected at random. All samples were homogenized at room temperature and stored at −20 °C before analysis.

#### 3.2.2. Collection and Preparation of Field Test Samples

In accordance with the strawberry sample collection requirements, strawberries of consistent growth maturity were randomly sampled at no less than 12 sampling points throughout the strawberry plots. Each strawberry sample collected in each plot should not be less than 1 kg. The strawberry samples were then homogenized. All the homogenized samples were sealed and stored at −20 °C.

### 3.3. Sample Pretreatment

Strawberry samples were pretreated using the QuEChERS method, as in our previous work [33]. A 10 g strawberry sample was placed in a 50 mL centrifuge tube. A total of 10 mL of acetonitrile was added into the centrifuge tube, and vortexed for 1 min. A total of 4 g of MgSO_4_ and 1 g of NaCl were added to the sample, which was vortexed for 1 min. The sample was vibrated mechanically for 30 min, and then, centrifuged for 5 minutes at 5000 rpm. An amount of 1 mL of supernatant was sampled, and d-SPE adsorbent (50 mg PSA, 50 mg C18, 150 mg MgSO_4_) was added to the samples, which were vortexed for 1 min and centrifuged at 10,000 rpm for 3 min. The supernatant was passed through a 0.22 μm filter membrane (Acrodisc^®^ syringe filter with GHP membrane, 13 mm) and transferred to a 2 mL microvial for UPLC-MS/MS analysis.

### 3.4. Analytical Conditions for UPLC-MS/MS

UPLC-MS/MS analysis was performed using a Waters UPLC system interfaced with a triple quadrupole mass spectrometer (TQS). An ACQUITY UPLC HSS T3 column (2.1 mm × 100 mm, 1.8 μm) was selected for the analysis. Water + 5 mM ammonium acetate (A) and acetonitrile (B) were used as the mobile phase with the following gradient program. Initially, 20% B at 0 min, 100% B at 3 min, 100% B at 5 min, 20% B at 5.5 min, and20% B at 7 min. Flow rate, column temperature, and injection volume were 0.3 mL min^−1^, 40 ℃, and 5 μL, respectively. The TQS was operated in positive and negative ion modes, with the following settings: ESI+/ESI− of 3 kV/1 kV; source temperature, 150 °C; desolvation temperature, 400 °C; flow of desolvation gas, 800 L h^−1^. Optimized multiple-reaction monitoring (MRM) mode was chosen to detect target compounds. The acquisition parameters are summarized in Table S1.

### 3.5. Method Validation

The linearity, accuracy, limit of detection (LOD), limit of quantification (LOQ), and precision of the UPLC-MS/MS method were validated according to European Commission SANTE/11312/2021 [27]. Linearity was determined at five concentrations (0.001–0.1 mg kg^−1^) in triplicate. Accuracy was determined in the recovery experiments using five replicates at three (Acrodisc^®^ syringe filter with GHP membrane, 13 mm) fortification levels. LOD was defined as the concentration of analyte detectable at a signal-to-noise ratio of 3:1. LOQ was estimated as the lowest fortified concentration yielding suitable recovery and precision. Precision was expressed as the relative standard deviation (RSD) of five replicate analyses on the same day, while within-laboratory reproducibility was determined on five consecutive days by three operators at three fortification levels.

### 3.6. Statistical Analysis

#### 3.6.1. Calculation of Prochloraz Concentration

The concentration of prochloraz is equal to the sum of the residues of prochloraz, BTS44596, and BTS44595 according to the definition of the European commission [34].

#### 3.6.2. Dissipation of Prochloraz

Strawberry samples were collected at specified sampling times after the spraying of prochloraz. Then, prochloraz residue was determined in the samples. The dissipation behavior of prochloraz was described using the first-order kinetic equation:

(1) $$C_{t}=C_{0}e^{-kt}$$

where

- $C_{t}$ is the residue concentration of prochloraz at time *t* (d);
- $C_{0}$ is the initial deposition of prochloraz (μg kg^−1^);
- *k* is the dissipation rate constant (d^−1^).

The dissipation half-life of prochloraz was obtained from:

T_1/2_ = ln2/*k* (2)

#### 3.6.3. Risk Assessment of Prochloraz Exposure

Risk assessment was calculated using two different approaches. In the 1st approach, the THQ method was used to evaluate the health risk of prochloraz according to the following equations.

The estimated daily intake (EDI) of pesticide was expressed as milligrams of pesticide intake per kilogram of body weight per day by eating strawberries (mg kg^−1^ d^−1^). EDI was calculated using the following formula:

(3) EDI=C×F/BW

where

*C* is the residue concentration in strawberry samples (mg kg^−1^).

F is the average daily fruit intake per person (kg person^−1^ d^−1^). The average daily fruit intake for children and adults was 80.4 and 64 g d^−1^, respectively [35].

BW is the average body weight (kg). The average weights of children and adults were 32.7 and 55.9 kg, respectively [36].

The target hazard quotient (THQ) was calculated using Equation (4):

(4) THQ=EDI/ADI

The ADI value is 0.01 mg kg^−1^, which refers to the regulation on prochloraz in the GB 2763-2021 National Food Safety Standard Maximum Residue Limit of Pesticides in Food [37]. If the THQ was <1, there was no obvious health risk to the exposed individuals; if the THQ was ≥1, it would indicate the possibility of a health risk for the exposed population.

In the 2nd approach, a standard deterministic risk assessment model, EFSA PRIMo revision 3.1, was applied to estimate the health risks for acute/chronic dietary exposure to pesticides [38]. In this approach, the acute and chronic exposure for consumers were calculated and compared with the acute reference dose (ARfD) and the acceptable daily intake (ADI), respectively. When the evaluated consumer exposure values did not exceed 100% of the ARfD or ADI, it indicated that the health risk was acceptable.

## 4. Conclusions

A simple, sensitive, and efficient method that combines the QuEChERS method and UPLC-MS/MS to simultaneously and quantitatively determine the residues of prochloraz and its metabolites in strawberries was established in this study. The proposed method with high recovery and accuracy is suitable for monitoring prochloraz and its metabolites in strawberries from a greenhouse. Prochloraz and its metabolites in strawberries were rapidly degraded following first-order kinetics models, with dissipation half-lives of 8.06 days under greenhouse conditions. The health risk associated with prochloraz in strawberries was evaluated using the THQ method and EFSA PRIMo model. In the risk assessment, the THQ values, %ARfD values, and %ADI values of Chinese children and adults were less than 1, indicating that no obvious health concerns of prochloraz are associated with the consumption of the studied strawberries. However, this study only investigated the dissipation of prochloraz and its metabolites in strawberries. Farmers may use multiple pesticides to control strawberry diseases. Therefore, the dissipation and risk assessment of prochloraz combined with other pesticides used in strawberries should be studied further.

## Figures and Tables

**Table 1:** Linearity and detection limit of prochloraz and its metabolites (*n* = 3).

Chemicals	Range (μg kg^−1^)	Linear Equation	R^2^	LOD (μg kg^−1^)
Prochloraz	1–60	Y = 89,410.8x + 448,897	0.9987	0.1
BTS44595	1–60	Y = 63,166.9x + 313,676	0.9983	0.1
BTS44596	1–100	Y = 5050.07x + 103,327	0.9972	0.1
BTS45186	5–100	Y = 97.2721x + 542.628	0.9969	1

**Table 2:** The spiked recoveries and RSDs of prochloraz and its metabolites in strawberries.

Chemicals	Spiked Level (μg kg^−1^)	Recovery (%)	Repeatability, RSD (%)	Within-Laboratory Reproducibility, RSD (%)
Prochloraz	10	116.01	3.53	4.19
	50	86.33	2.89	5.31
	100	94.93	4.24	5.26
BTS44595	10	73.06	2.24	4.27
	50	111.42	1.12	3.57
	100	105.4	3.12	6.15
BTS44596	10	96.01	5.79	5.28
	50	113.67	3.49	6.37
	100	90.27	1.74	5.76
BTS45186	10	108.11	8.76	5.93
	50	94.67	2.54	6.31
	100	90.53	6.62	9.17

**Table 3:** Dietary intake assessment of prochloraz in strawberries under greenhouse conditions.

Individuals	ADI (mg kg^−1^ b.w. day^−1^)	EDI (mg kg^−1^·d^−1^)	THQ	%ARfD	%ADI	Health Risk
Children	0.01	2.0 × 10^−4^	2.0 × 10^−2^	4.22	2.50	No
Adults		1.5 × 10^−4^	1.5 × 10^−2^	3.71	1.16	No

## Data Availability

Data are contained within the article and Supplementary Material.

## Supplementary Materials

The following supporting information can be downloaded at: https://www.mdpi.com/article/10.3390/molecules28227498/s1, Table S1. UPLC-MS/MS parameters for prochloraz and its metabolites. Figure S1. MRM images of prochloraz and its metabolites separated by different chromatographic columns. (a) Matrix standard at 30 μg L^−1^ was separated using ACQUITY UPLC HSS T3 column; (b) matrix standard at 50 μg L^−1^ was separated using ACQUITY UPLC BEH C18. Figure S2. MRM images of prochloraz and its metabolites (50 μg L^−1^) eluted by different mobile phases: (a) ultrapure water (A), methanol (B); (b) ultrapure water + 5 mM ammonium formate (A), methanol (B); (c) ultrapure water + 5 mM ammonium acetate (A), methanol (B); (d) ultrapure water (A), acetonitrile (B); (e) ultrapure water + 5 mM ammonium formate (A), acetonitrile (B).
