# Supplementary material for: Dissipation and Dietary Risk Assessment of Prochloraz in Strawberries under Greenhouse Conditions
Source: Molecules. 2023 Nov 9;28(22):7498. doi: 10.3390/molecules28227498 (PMC10672735; doi:10.3390/molecules28227498)
Supplement: Supplementary file 1 [file molecules-28-07498-s001.zip › molecules-2608328-supplementary.pdf]

Supplementary information

Dissipation and Dietary Risk Assessment of Prochloraz in Strawberries under Greenhouse Conditions

Table S1. UPLC-MS/MS parameters for prochloraz and its metabolites.

Pesticide	Molecular formula	Retention time (min)	Precursor ion (m/z)	Quantification transition (m/z)	Cone voltage (v)	Collision energy (ev)	Confirmatory transition (m/z)	Cone voltage (v)	Collision energy (ev)
Prochloraz	$C_{15}H_{16}Cl_3N_3O_2$	3.47	376.2	308.1	18	16	70.1	18	34
BTS44595	$C_{12}H_{15}Cl_3N_2O_2$	3.17	325.1	129.1	44	16	86.1	44	20
BTS44596	$C_{13}H_{15}Cl_3N_2O_3$	3.37	353.0	70.1	12	24	256.9	12	16
BTS45186	$C_6H_3Cl_3O$	2.87	194.9	159.0	20	22	34.0	20	20

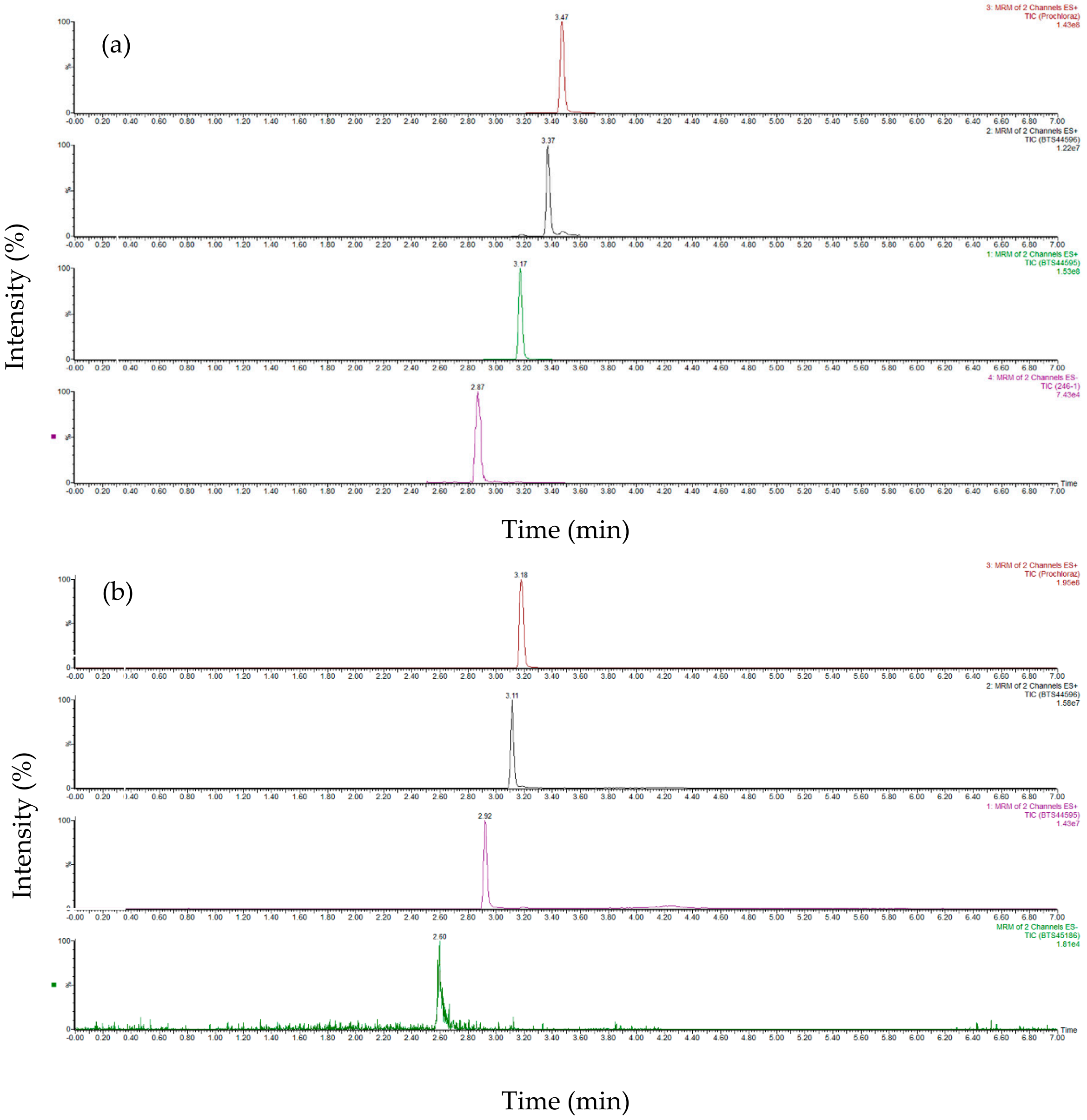

Figure S1. MRM images of prochloraz and its metabolites separated by different chromatographic columns. (a) Matrix standard at 30 μg L^{-1} were separated by ACQUITY UPLC HSS T3 column; (b) Matrix standard at 50 μg L^{-1} were separated by ACQUITY UPLC BEH C18.

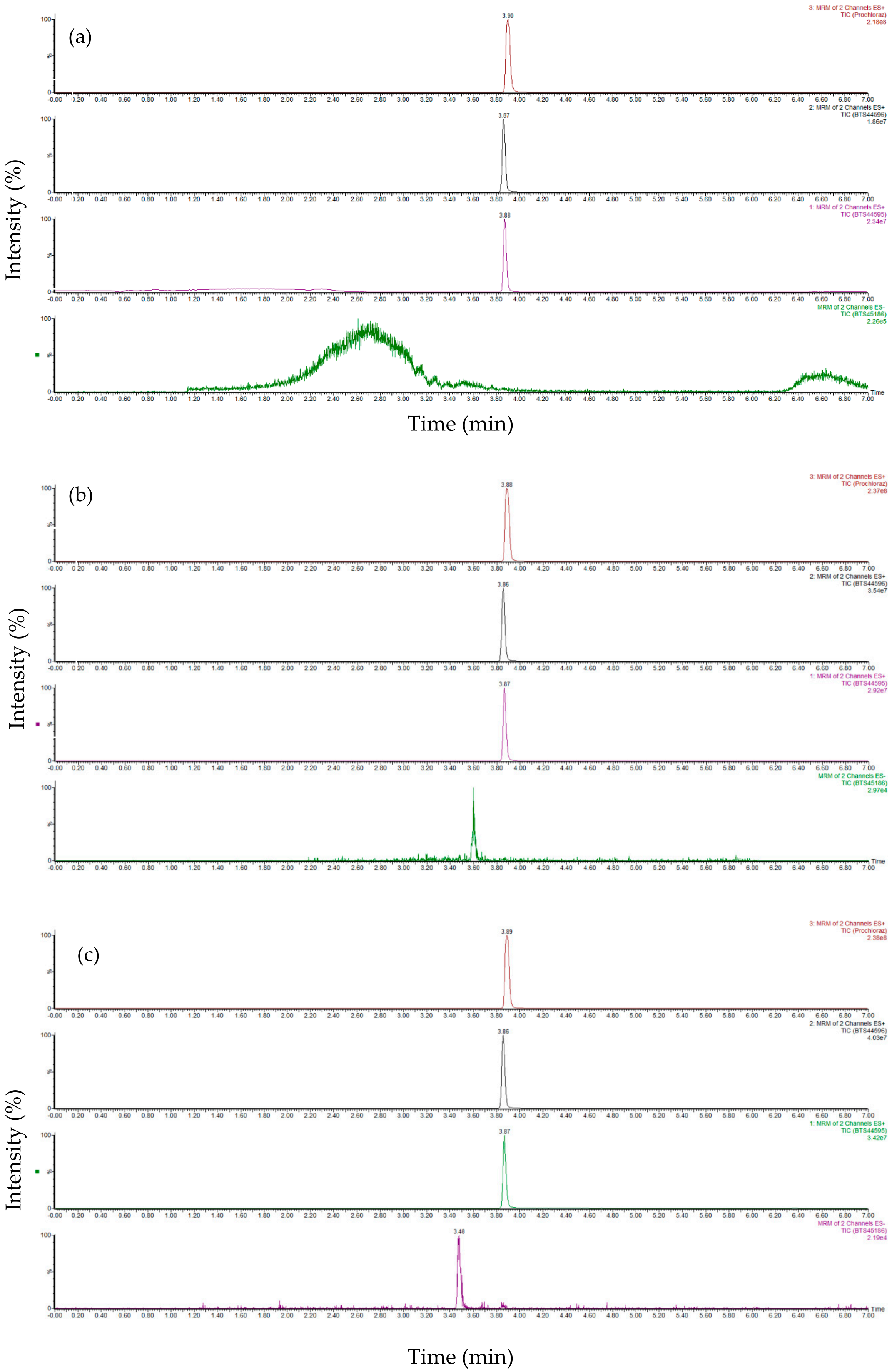

(a)
3: MRM of 2 Channels ES+
TIC (Prochloraz)
2.18e8
2: MRM of 2 Channels ES+
TIC (BTS44596)
1.86e7
1: MRM of 2 Channels ES+
TIC (BTS44595)
2.34e7
MRM of 2 Channels ES-
TIC (BTS45186)
2.26e5
(b)
3: MRM of 2 Channels ES+
TIC (Prochloraz)
2.37e8
2: MRM of 2 Channels ES+
TIC (BTS44596)
3.54e7
1: MRM of 2 Channels ES+
TIC (BTS44595)
2.92e7
MRM of 2 Channels ES-
TIC (BTS45186)
2.97e4
(c)
3: MRM of 2 Channels ES+
TIC (Prochloraz)
2.38e8
2: MRM of 2 Channels ES+
TIC (BTS44596)
4.03e7
1: MRM of 2 Channels ES+
TIC (BTS44595)
3.42e7
MRM of 2 Channels ES-
TIC (BTS45186)
2.19e4
Intensity (%)
Time (min)

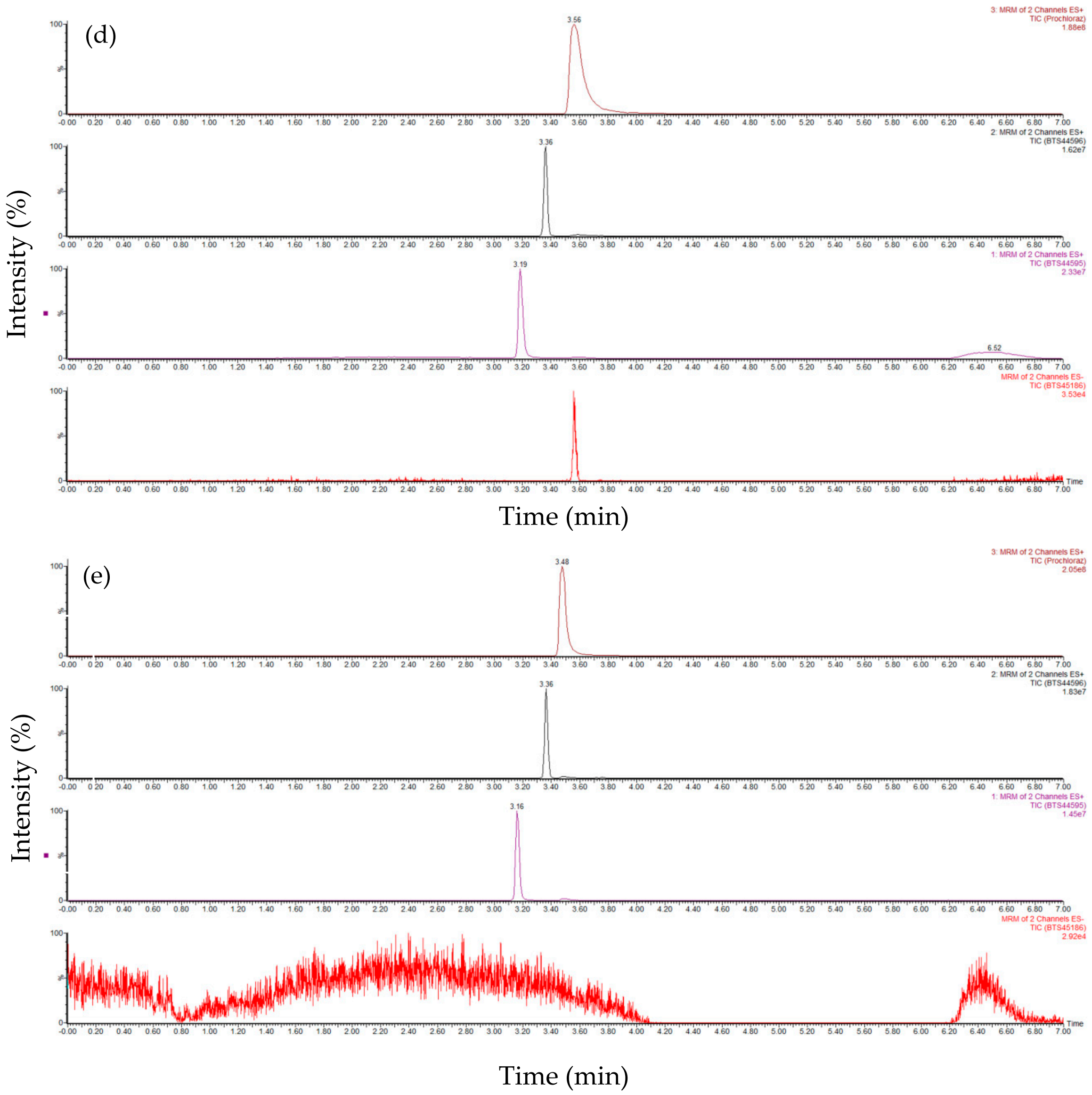

Figure S2. MRM images of prochloraz and its metabolites eluted by different mobile phases (50 μg L^{-1}). (a) ultrapure water (A), methanol (B); (b) ultrapure water+5 mM ammonium formate (A), methanol (B); (c) ultrapure water+5 mM ammonium acetate (A), methanol (B); (d) ultrapure water (A), acetonitrile (B); (e) ultrapure water +5 mM ammonium formate (A), acetonitrile (B).
